# Pharmacokinetics of Linezolid and Ertapenem in experimental parapneumonic pleural effusion

**DOI:** 10.1186/1476-9255-7-22

**Published:** 2010-05-18

**Authors:** Maria Saroglou, Stavros Tryfon, Georgios Ismailos, Ioannis Liapakis, Manolis Tzatzarakis, Aristidis Tsatsakis, Apostolos Papalois, Demosthenes Bouros

**Affiliations:** 1 1st Pulmonary Clinic, General Hospital «G Papanikolaou», Thessaloniki, Greece; 2 Experimental & Research Center Elpen Pharmaceutical Co, Athens, Greece; 3 Department of Pneumonology, Medical School, Democritus University of Thrace, Alexandroupolis, Greece; 4 Center of Toxicological Science and Research, Dept of Medicine, University of Crete, Heraklion, Greece

## Abstract

**Objective:**

To determine the extent of linezolid and ertapenem penetration into the empyemic fluid using a rabbit model of empyema.

**Methods:**

An empyema was created via the intrapleural injection of *Escherichia coli *bacteria (ATCC 35218) into the pleural space of New Zealand white rabbits. After an empyema was verified by thoracocentesis, 24 hours post inoculation, linezolid (10 mg/kg) and ertapenem (60 mg/kg) were administered intravenously into 10 and 8 infected empyemic rabbits, respectively. Antibiotic levels were determined in samples of pleural fluid and blood serum, collected serially at 1, 2, 4, 6 and 8 hours, after administration each of the two antibiotics.

**Results:**

Linezolid as well as ertapenem penetrate well into the empyemic pleural fluid, exhibiting a slower onset and decline compared to the corresponding blood serum levels. Equilibration between blood serum and pleural fluid compartments seems to occur at 1.5 hours for both linezolid and ertapenem, with peak pleural fluid levels (Cmax_pf _of 2.02 ± 0.73 «mu»g/ml and Cmax_pf _of 3.74 ± 1.39 «mu»g/ml, correspondingly) occurring 2 hours post antibiotics administration and decreasing very slowly thereafter. The serum concentrations for both antibiotics were significantly lower from the corresponding pleural fluid ones during the 8 hours collecting data, with the exception of samples collected at the 1^st ^hour (Cmax_serum _of 2.1 ± 1.2 «mu»g/ml for linezolid and Cmax_serum _of 6.26 ± 2.98 «mu»g/ml for ertapenem).

**Conclusion:**

Pleural fluid levels of both antibiotics are inhibitory for common specified pathogens causing empyema.

## Introduction

The annual incidence of bacterial pneumonia is estimated to be 2-4 million in the USA, with approximately 20% of patients requiring hospitalization [1]. Of these patients 40-60% develop parapneumonic pleural effusions and pleural empyemas occur in 5-10% [2]. Mortality among patients with thoracic empyema ranges from 5 to 30% [3] and if the patients are immunocompromised it can be as high as 40% [2].

The bacteriology of pleural empyema is related to that of the pneumonic infection and is usually due to a mixture of anaerobic and aerobic organisms. Streptococci (*Streptococcus pneumoniae)*, and staphylococci (*Staphylococcus aureus) *usually dominate gram-positive isolates, while *Escherichia coli*, *Klebsiella *species, *Pseudomonas *species, and *Haemophilus influenzae *are the most common gram-negative isolates [4]. Anaerobic organisms are often found in combination with other organisms.

There are several therapeutic options available [5] and the choice of therapy is usually dictated by the severity of the disease on presentation and should follow the existing guidelines for treatment of community or hospital-acquired pneumonia [2]. The usual initial treatment remains parenteral antibiotics with chest tube placement [6]. Therapy needs to be initiated as soon as pleural fluid, sputum and blood samples have been taken but with the following in mind: in addition to the specificity of antimicrobial agent for the offending microorganism, its distribution within the body is a critical factor in determining its therapeutic efficacy. If the antimicrobial agent does not enter the side at which the offending microorganism resides, bacterial growth will continue despite *in vitro *susceptibility of the organism to the drug. In fact, specific to the treatment of pleural infections, it is important to obtain sufficient levels of the antibiotics in the pleural fluid [7]. Second and third generation cephalosporins, beta-lactam-beta-lactamase inhibitor combinations, fluoroquinolones, metronidazole, clindamycin, meropenem, or aztreonam may be considered [8].

The purpose of the present study was to determine the pharmacokinetic parameters of linezolid and ertapenem in the blood and pleural fluid in an experimental rabbit model of empyema, after intravenous administration. It was hypothesized that both antibiotics would penetrate well into the pleural fluid and achieve therapeutic levels in the pleural fluid of rabbits with empyema.

## Materials and methods

### Animals

A total of 22 male New Zealand white rabbits (weight range 2.40-2.70 kg) were used for the study. The animals were housed in individual cages and allowed food and tap water *ad libitum*. Room temperature ranged between 18 and 22°C, relative humidity between 55 and 65% and the light/dark circle was 6 am/6 pm. The study protocol was approved by the Veterinary Administration Medical Centre, Athens, Greece, in conformance to the 160/1991 Council Directive of the EU.

### Bacteria preparation

The *Escherichia coli *strain (ATCC 35218) was grown on McCongi agar (Becton Dickinson, Sparks, MD, USA) for 24 h at 35°C. *Escherichia coli *bacteria (in a 5-ml volume of saline solution) were injected into the right pleural space.

### Empyema induction

The rabbits were anaesthetised with ketamine; 50 mg/kg i.m. (Ketaset; Fort Dodge Laboratories Inc., Fort Dodge, IA, USA); atropine, 0.04 mg/kg (Demo SA, Athens, Greece); and xylazine, 5 mg/kg i.m. (Rompun; Bayer AG, Leverkusen).

The right chest wall of each rabbit was shaved and then scrubbed with povidone-iodine and alcohol. The animals were placed in a supine position on an operating table, under a heating lamp, and a 0.5-cm medial-to-lateral skin incision was made over the right anterior chest, using a scalpel. A specially prepared 16-gauge angiocatheter (containing additional holes near the tip of the catheter) was then introduced into the pleural space. After the placement of the catheter, any air within the pleural space was aspirated and the catheter was secured s. c. in the area between the scapulas [9]. The chest tubes were attached to a Heimlich valve with a three-way stopcock, in-line between the chest tube and the Heimlich valve. Turpentine (1 ml) [10] (Riedel de Haen; Sigma-Aldrich Laborchemikalien, GMBH, Seelze Germany) was administered into the pleural space of the animals and the chest tube was then flushed with 1.5 ml of saline solution. *Escherichia coli *(ATCC 35218 in a final volume of 5 ml saline) were injected 24 h later through the cannula into the pleural cavity of the animals.

### Empyema verification

A maximum of 0.5 ml of pleural fluid was removed for analysis 2, 8, 24, 48, and 72 hours, after bacterial injection. The pH of the pleural fluid was estimated using a blood gas machine [11] (Gem premier 3000, model 5700; Instrumentation laboratory, Lexington, MA, USA), and the glucose and lactate-D-hydrogenase (LDH) levels, were estimated using a common microbiological laboratory test (the upper limit of LDH in the present study's laboratory was 460 U/L). An empyema was said to be present if the pleural fluid (selected appeared grossly infected, if the glucose levels were < 40 mg/dl and if the pleural fluid pH was < 7.10 and the LDH was > 1,000 U/L [12]. Data of rabbits' pleural fluid biochemical analysis are presented on table 1.

**Table 1:** Pleural fluid analysis in experimental rabbits model. 24 hours after bacterial inoculation (*Escherichia coli*).

Animal	pH	Glucose (mg/dl)	LDH (U/L)
Control 1	7.083	17	2025

Control 2	7.099	20	3113

Control 3	7.084	24	1875

Control 4	6.950	18	2296

**Ertapenem group**			

1	6.780	8	3113

2	6.932	11	8307

3	7.036	10	9180

4	6.862	9	3188

5	6.900	6	2070

6	7.160	11	4400

7	7.031	12	2248

8	7.009	18	2902

**Linezolid group**			

1	6.907	10	9180

2	7.080	22	4540

3	7.090	19	2574

4	6.700	9	2877

5	7.100	19	4030

6	7.090	22	4540

7	6.914	13	12500

8	7.100	24	2902

9	7.080	20	2248

10	7.000	22	4400

### Antibiotic administration

After the presence of an empyema was verified, 10 rabbits were injected with linezolid, (10 mg/kg) [Zyvoxid 600 mg/vial i.v., Pfizer, Italy], and 8 with ertapenem (60 mg/kg) [Invanz 1gr/vial i.v., Merck Sharp & Dohme, USA] through their marginal ear vein over a 15-min period. Four animals served as controls and were infected with *Escherichia coli *but were not treated with any antibiotic.

### Pleural fluid and blood specimens

Blood and empyemic pleural fluid specimens were serially collected 1, 2, 4, 6 and 8 hours, after administration of each antibiotic, for the estimation of antibiotic levels. Immediately after the specimens were collected, the blood and the pleural fluid samples were centrifuged at 3,000 rpm for 15 min. Supernatants were then refrigerated at -20°C overnight. Duplicate specimens of blood and pleural fluid were obtained at each time point. The means of the duplicate values at each time point were used for the analysis.

### Sample preparation and antibiotic level estimation

#### Chemicals

Methanol and acetonitrile (Hiper Solv BDH Laboratory supplies Poole, BH15 1TD, England), water and orthophosphoric acid 85%, (E. Merck, Darmstadt, Germany) were used for the HPLC analysis.

#### Preparation of standard curves

Stock solutions of linezolid and ertapenem were prepared in 100 μg/ml of water and stored at 4°C. Eight point standard curves for linezolid and ertapenem were prepared at concentration 0, 0.125, 0.25, 0.5, 1, 2.5, 5 and 10 μg/ml. The curves were linear for both drugs with coefficients of linearity greater than 0.999.

#### Quantification of biological extracts

Blood samples from healthy animals were used as blank samples. An 8-point calibration curve was prepared for both drugs. The final concentrations of the fortified samples were 0, 0.25, 0.5, 1, 2.5, 5, 10 and 25 μg/ml. The calibration curves were linear (r^2 ^= 0.9983, for linezolid and r^2 ^= 0.9966 for ertapenem). The limit of quantification (LOQ) were calculated on the basis of signal to noise ratio 10, (S/N = 10) and was determined at 0.125 μg/ml and 0.25 μg/ml for linezolid and ertapenem respectively.

### Apparatus

The quantification of the samples was performed by a reversed-phase HPLC method (on a Spectra Physics 8800, San Jose, California, USA) using a Supelco, 5 μm, 250 mm × 4.6 mm ID column (Discovery C18, 595 North Harrison Road, USA). The mobile phase for the determination of linezolid consisted of acetonitrile:water (20:80 v/v) [13] and water:methanol:orthophosphoric acid (64:35:1 v/v/v) for ertepenem [14]. The elution conditions were isocratic and the mobile phase flow-rate was set at 1.0 ml/min and 0.8 ml/min for linezolid and ertapenem respectively. UV absorbance detection at 254 nm (linezolid) and 298 nm (ertapenem) was carried out with a uv/vis detector (Spectra Physics 8450, San Jose, California, USA) with the range set at 0.01 AUFS. Under these conditions the retention time of linezolid and ertapenem was 13.7 min and 15.7 min respectively.

### Extraction procedure for HPLC analysis

The biological sample (100 μl), (blood or pleural empyema) was diluted with 100 μl of acetonitrile (for linezolid) [15] or 100 μl of methanol (for ertapenem) [14]. The solution was vortexed for 20 sec and centrifuged for 5 min at 14000 rpm in order to separate the precipitated proteins; 20 μl of the supernatant was injected into the column. The recovery of linezolid and ertapenem was calculated at 102.5% and 104.1% respectively by spiked solutions at a concentration of 0.5, 1, 5, 10 μg/ml.

### Necropsy of the rabbits

The antibiotic-free rabbits from the control group died 24 to 48 hours after the inoculation, with fever and diarrhea. The rabbits in which, ertapenem and linezolid were infused, were sacrificed via a lethal dose of pentobarbital i.v. through the marginal ear vein after the collection of the 8^th ^hour sample.

### Statistical analysis

All data were entered into Microsoft Office, Excel, and analyzed using the statistical program SPSS (13.0 Illinois, Chicago). Analysis was carried out by means of Student's t-tests with a p < 0.05 level considered statistically significant. The areas under the concentration-time curves (AUC) were estimated by the trapezoidal rule, while the terminal half-life values were estimated from two or three time-points, corresponding to the antibiotic's elimination phase.

## Results

All rabbits developed empyema after the intrapleural injection of turpentine and the inoculation with *E. coli*. In all cases, and in accordance to previously reported data [9] the estimated pleural fluid pH was < 7.10, while the corresponding glucose levels were < 30 mg/dL (Table 1).

### Linezolid

**Figure 1:**
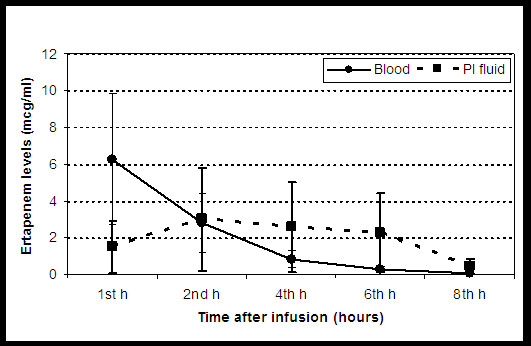
**Linezolid concentrations (mean ± sd, mcg/mL) in blood serum (circles) and pleural fluid (squares), in an experimental rabbit model of pleural empyema induced by inoculation with Escherichia coli (ATCC 35218), after the i.v. administration of an linezolid solution (10 mg/kg) to 10 New Zealand white rabbits**.

The mean (SD) linezolid concentration values for both blood serum and pleural fluid samples (Cserum and Cpf, respectively) corresponding to the sampling time intervals (1, 2, 4, 6, 8 hours post administration) are shown in Figure 1 and Table 2. The area under the concentration versus time curve (AUC) was almost 4-fold higher in the pleural fluid compared to the blood serum compartment. The terminal half-life (T_1/2_) of linezolid was equal for the two compartments, with a slight superiority for the T_1/2 _of the pleural fluid (Table 3). The time to equilibration between the pleural fluid and blood serum compartments seems to occur at 1.5 hours, with an onset of approximately 60% at the first hour compared to the corresponding blood serum levels. The peak pleural fluid levels (Cmax_pf _of 2.02 ± 0.73 μg/ml) occurring 2 hours post administration and decreasing slowly thereafter. With the exception of samples collected at 1 hour (Cmax_serum _of 2.10 ± 1.20 μg/ml versus Cmax_pf _of 1.28 ± 1.60 μg/ml), linezolid serum concentration values were significantly lower from the corresponding pleural fluid ones (Student's t-test, p < 0.05), during the 8 hours data collecting period (Figure 1).

**Table 2:** Mean (± sd) concentrations (mcg/ml) of linezolid and ertapenem in blood serum (Cserum) and pleural fluid (Cpf) after the iv administration of a linezolid solution (10 mg/kg infused over a period) and an ertapenem solution (60 mg/kg infused over a period), to 10 and 8 New Zealand white rabbits, respectively

	1 h	2 h	4 h	6 h	8 h
Linezolid					

Cserum	2.10 ± 1.20	0.20 ± 0.28	< QL	< QL	< QL

N	10	10	9	7	7

Cpf	1.28 ± 1.60	2.02 ± 0.73*	1.70 ± 1.00	0.79 ± 0.85	0.16 ± 0.25

N	10	9	9	7	6

Ertapenem					

Cserum	6.26 ± 2.98	2.80 ± 1.64	0.97 ± 1.09	0.79 ± 0.85	0.39 ± 0.25

N	8	8	8	7	6

Cpf	3.00 ± 1.55*	3.74 ± 1.39*	3.62 ± 1.40*	3.16 ± 1.20*	2.84 ± 0.90*

N	8	8	7	7	5

**N **= number of experimental animals, **QL**: estimations are lower than the analytical quantification limit. *: significantly different values from the corresponding pleural fluid ones (p < 0.05. student's t-test).

**Table 3:** The basic pharmacokinetic parameters of linezolid and ertapenem in pleural fluid and in blood serum after the i.v. infusion of linezolid solution (10 mg/kg) and of ertapenem solution (60 mg/kg) to 10 and 8 New Zealand white rabbits, respectively.

	Blood serum	Pleural fluid	R_pf/ser_
**Linezolid**			

**AUC _0-8 h _**(h.mcg/mL)	2.20	9.45	4.29

**AUC _0-inf _**(h.mcg/mL)	2.24	9.72	4.34

**T_1/2 _**(hours)	0.98	1.18	

**Ertapenem**			

**AUC _0-8 h _**(h.mcg/mL)	12.70	17.74	1.39

**AUC _0-inf _**(h.mcg/mL)	12.77	18.76	1.47

**T_1/2 _**(hours)	1.03	1.49	

**AUC _0-8 h/0-inf _: **area under the linezolid/ertapenem concentration versus time curve corresponding to 0-8 hours and 0-inf time intervals, respectively. **T_1/2_**: terminal elimination half-life, of linezolid/ertapenem in pleural fluid and in blood serum. **R_pf/ser _**: linezolid/ertapenem penetration ratio into the pleural fluid compared to blood serum.

### Ertapenem

**Figure 2:**
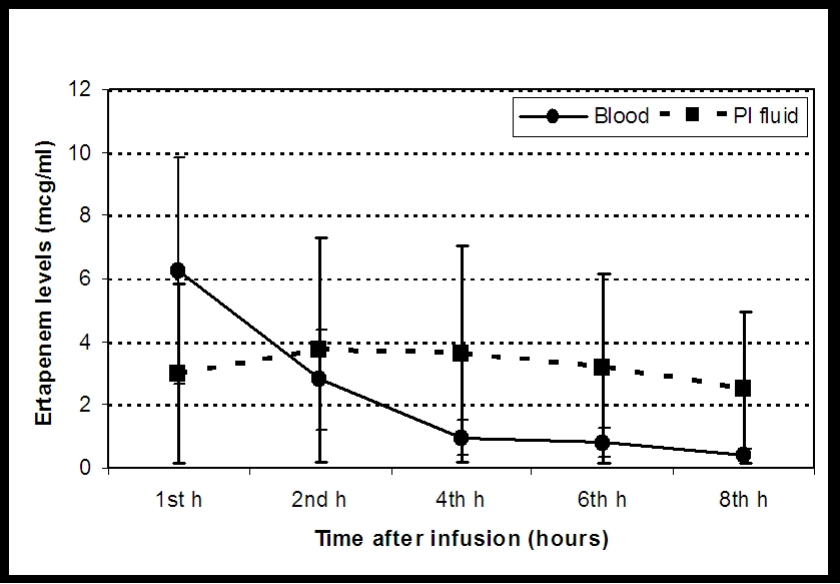
**Ertapenem concentrations (mean ± sd, mcg/mL) in blood serum (circles) and pleural fluid (squares), in an experimental rabbit model of pleural empyema induced by inoculation with Esherichia coli (ATCC 35218), after the i.v. administration of an ertapenem solution (60 mg/kg) to 8 New Zealand white rabbits**.

The mean (SD) ertapenem concentration values for both blood serum and pleural fluid samples (Cserum and Cpf, respectively) corresponding to the sampling time intervals (2, 8, 24, 48 and 72 hours post administration) are shown in Figure 2 and Table 2. The area under the concentration versus time curve (AUC) and terminal half-life (T_1/2_) of ertapenem was approximately one and half higher in the pleural fluid compared to the blood serum compartment (Table 3). Ertapenem penetrated into the empyemic pleural fluid, exhibiting a similar onset and a slightly lower decline compared to the corresponding blood serum compartment (T_1/2 serum _= 1.03 vs T_1/2 pf _= 1.49). Equilibration between pleural fluid and blood serum seems to occur at 1.5 hours after the ertapenem administration, along with a concentration of 2.8 μg/ml. With the exception of samples collected at 1 hour (Cmax_serum _of 6.26 ± 2.98 μg/ml versus Cmax_pf _of 3 ± 1.55 μg/ml), ertapenem serum concentration values were significantly lower than the corresponding pleural fluid ones (Student's t-test, p < 0.05), during the 8 hours data collecting period (Figure 2). There is a limitation of the above estimations of the serum concentration of linezolid because these made by only two time points measurements.

## Discussion

The present study shows that linezolid and ertapenem penetrated well into empyemic pleural fluid. Significant levels of both antibiotics remained in the pleural space for up to 4 hours, despite diminishing blood antibiotic levels over time. Specifically for ertapenem the elevated pleural drug levels remained higher for the 8-hour period of our study. These findings suggest that both antibiotics show elevated concentrations and extended terminal half-life time into the pleural cavity compared to the serum. These facts, combined with the high extracellular distribution of these antibiotics, suggest that they may be effective in the treatment of pleural bacterial infection.

There are limited number of articles in the literature, which have studied the correlation between the pleural fluid and serum antibiotic levels. Teixeira et al [16] using a rabbit model of empyema, determined the relationships between the pleural fluid and serum antibiotic levels of metronidazole, penicillin, clindamycin, ceftriaxone, vancomycin and gentamicin. The pharmacokinetics of these drugs were studied following intravenous administration. It was found that the penetration of these antibiotics into the infected pleural fluid and the equilibration between the blood serum and pleural fluid varied substantially between the various antibiotics. Liapakis et al studied the relationship between the pleural fluid and blood serum antibiotic levels of clarithromycin [17] levofloxacin and moxifloxacin [18]. We used the same rabbit model and protocol for detecting the relationship between linezolid and ertapenem levels in blood serum and pleural fluid.

Linezolid is a novel oxazolidinone antimicrobial drug, with an enhanced in vitro activity against Gram-positive pathogens. It is a protein synthesis inhiditor [19] that stops translation at the initiation step that involves the binding of N-formylmethionyl-tRNA to the 70S ribosome [20].

There are studies demonstrating its efficacy against aortic valve endocarditis [21], staphylococcal endocarditis [22] in patients with community-acquired and nosocomial pneumonia [23], and its comparative efficacy versus other antibiotics in patients with pneumonia [24,25]. The intrapulmonary pharmacokinetics of the drug has been thoroughly studied [26]. There is a case report for successful treatment with linezolid of meningitis [27] and data about its ability to penetrate into the cerebrospinal fluid [28]. However, there are no studies on linezolid pharmacokinetics into the pleural effusion. Our study shows that linezolid penetrates in the pleural fluid exhibiting a 4-fold higher concentration level compared to the blood serum compartment and an equal elimination half - time (T_1/2_).

Ertapenem, a new carbapenem, demonstrates a broad spectrum of in vitro activity against most Gram-negative and Gram-positive organisms associated with community-acquired and hospital-acquired infections [29]. Compared to other carbapenems it has a relatively long half-life due to high protein binding capacity, permitting once daily administration [30]. The in vitro activity of ertapenem is retained against most isolates that produce high-level AmpC βeta-lactamases (cephalosporinases) and clavulanic-acid-inhibited extended-spectrum βeta-lactamases [31]. There are several studies exhibiting its efficacy in patients with complicated intra-abdominal infections [32], urinary tract and pelvic infections [33], and community-acquired pneumonia [34]. There is a study concerning for the penetration of ertapenem into the inflamed and non-inflamed meninges [35], while the penetration in the pleural effusion has been studied for the other antibiotic agents of the carbapenem family (imipenem, meropenem, panipenem and biapenem) [36]. In this study, ertapenem penetrated well into the empyemic pleural fluid, exhibiting a similar onset and a slightly lower decline compared to the corresponding blood serum compartment. The AUC values and the elimination half-time (T_1/2_) of ertapenem was approximately one and half higher respectively in the pleural fluid compared to the blood serum compartment. Based on literature data that penetration of the meropenem in the pleural fluid [37] which belongs into the same family with ertapenem and the penetration of ertapenem into cerebrospinal fluid [36] we suggest that ertapenem may be effective in the treatment of pleural effusion.

The equilibration between an antibiotic in the serum and the pleural fluid depends on several factors. These include the thickness of the pleura (equilibration will occur less rapidly with a thicker pleura), the size of the pleural effusion (equilibration will occur less rapidly with larger pleural effusions), the degree of pleural inflammation (equilibration will occur more rapidly with inflammation both due to increased protein flux; and the infiltration of macrophages to the inflammatory area) and the antibiotic itself {molecular weight, presence or absence of liposolubility [38] and protein binding [39]}. In our study, higher pleural fluid levels of linezolid and ertapenem than serum levels were measured continuously, after the first two hours of intravenous administration of both drugs. It is not clear if these results obtained in the rabbit can be extrapolated exactly to humans, as rabbits are a species with thin visceral pleura, while humans have thick visceral pleura [40]. It is likely that the equilibration of antibiotics would be more rapid in species with thin pleura.

In conclusion, our data suggest that linezolid and ertapenem penetrated well into the epmyema pleural fluid, achieving concentrations 2 to 3 fold higher than those in the serum, for an extended period of time. Due to the above mentioned differences of pleura anatomy between rabbits and humans, in vivo studies are needed in patients in order to evaluate the exact penetration characteristics of these antibiotics into the human pleural fluid. A recent study found that the combination of linezolid with ertapenem was associated with in vitro and in vivo highly synergistic antimicrobial activity [41]. Based on this, the pharmacokinetic advantages of linezolid and ertapenem in the pleural fluid make them as promising therapeutic agents for parapneumonic pleural effusion, when they are administered alone or in combination.

## Competing interests

The authors declare that they have no competing interests.

## Authors' contributions and agreement

MS carried out the experimental protocol and collected all the data. ST and GI contributed in the design of the protocol, performed the statistical analysis, were involved in the drafting of the manuscript and carried out the revisions of the final version. MT and AT participated in the design of the study, carried out the analytical procedures and were involved in the drafting of the methodology of the manuscript. IL and AP participated in the design and the validation of the empyemic rabbit model. DB designed the experimental protocol and gave the final approval for the submission of this manuscript.

All authors read and approved the final manuscript.

This study was supported by a grant from Experimental Research Center ELPEN A. E. Farma, Athens, Greece.
